# Pre-breeding of lentil (*Lens culinaris* Medik.) for herbicide resistance through seed mutagenesis

**DOI:** 10.1371/journal.pone.0171846

**Published:** 2017-02-14

**Authors:** Muhammad Rizwan, Muhammad Aslam, Muhammad Jawad Asghar, Ghulam Abbas, Tariq Mahmud Shah, Hussein Shimelis

**Affiliations:** 1 Nuclear Institute for Agriculture and Biology, Faisalabad, Pakistan; 2 Department of Plant Breeding and Genetics, University of Agriculture Faisalabad, Faisalabad, Pakistan; 3 School of Agricultural, Earth and Environmental Sciences, African Centre for Crop Improvement, University of KwaZulu-Natal, Pietermaritzburg, South Africa; United States Department of Agriculture, UNITED STATES

## Abstract

Lentil is a poor competitor of weeds and its sensitivity to herbicides is a major hurdle for large scale production. The present study was conducted to select herbicide resistant lentil genotypes through seed mutagenesis. Seeds of three advanced lentil genotypes (LPP 11001, LPP 11100 and LPP 11116) were treated with two different concentrations of ethyl methanesulfonate (EMS; 0.1 and 0.2%), hydrazine hydrate (HH; 0.02 and 0.03%) and sodium azide (SA; 0.01 and 0.02%) to develop M_1_ seed. The M_2_ was screened against two herbicides including Ally Max 28.6% SG (X = 34.58 g/ha and 1.5X = 51.87 g/ha) and Atlantis 3.6% WG (X = 395.2 g/ha and 1.5X = 592.8 g/ha) using the following three screening methods: post plant emergence (PPE), pre-plant incorporation (PPI) and seed priming (SP). Data were recorded on survival index and survival percentage from each experimental unit of every population. Plants in all populations were categorized following their reaction to herbicides. The newly developed populations showed greater variation for herbicide resistance when compared to their progenitors. Phenotypic traits were significantly reduced in all the screening environments. Overall, 671 herbicide resistant mutants were selected from all testing environments. The seeds from selected plants were re-mutagenized at 150 Gy of gamma radiation and evaluated against higher dose of herbicides. This allowed selection of 134 herbicide resistant mutants. The selected mutants are useful germplasm for herbicide resistance breeding of lentil.

## Introduction

Lentil (*Lens culinaris* Medik.) is an important cool season pulse crop. The increase in productivity of this crop has resulted from cultivars which were developed through the genetic improvement of high seed yield and disease resistance. Some non-genetic, crop management practices have also been developed in the past for diseases and insect control through the use of chemical protectants. There is still a yield gap which exists due to inadequate weed control. Poor weed control due to the sensitivity of lentil to herbicides is a major hurdle for large scale production. The potential yield of present cultivars of lentil ranges between 1500 and 2000 kg ha^-1^ but the average yield (657 kg ha^-1^) is considerably low which is mainly due to poor weed management [1]. Lentil is regarded a poor competitor with weeds [2] which compete with lentil plants for plant nutrients, moisture, light, space and also deprive the crop plants of nitrogen thus causing a considerable loss in yield.

Genetic tolerance of lentil to herbicides has several benefits like increased safety margins between weed and crop sensitivity, expanded applicability of a particular herbicide and adequate weed control with lower operating cost as compared to manual weeding and crop rotation. Genetic manipulation of lentil to make it herbicide resistant could allow the use of herbicides with broader weed control spectrum. In addition, the compounds that have less residual effects, effective at low application rates and have favorable toxicological properties might become more useful with the removal of crop selectivity constraints. In view of these facts, it is very crucial to break sensitivity of lentil to herbicides and selection of herbicides targeting only weeds is difficult to achieve.

During the last five decades, various attempts were made to develop crop plants with tolerance to herbicide doses that would normally be fatal to the weeds. Different scientific approaches were used to genetically modify the crop plants for engineering herbicide tolerant/resistant crops. For instance, herbicide tolerant tobacco mutant plants were obtained through selection of cell lines in tissue culture [3]. This herbicide tolerance in tobacco mutants was linked with less sensitivity of acetolactate synthase (ALS) to sulfonylurea herbicides as compared with control plants [4]. Another approach was to bring genetic modification in crop plants through plant transformation with genes responsible for herbicide resistance [5]. Seed mutagenesis followed by screening and selection of herbicide resistant plants has been in practice [6]. Screening at whole plant level has also been in use to identify induced variants of tomato [7] and natural variants of soybean [8] conferring tolerance to herbicides.

Due to less responsiveness of leguminous crops to transformation, scientists have tried to develop imidazolinone and sulfonylurea resistant crops through seed mutagenesis. At present, considerable achievements have been made in this field [9]. Seed mutagenesis is an important tool to develop herbicide resistant crops and most of the herbicide tolerance traits were developed through chemical mutagenesis. Among chemical mutagens different mutagens such as ethyl methanesulfonate (EMS), sodium azide and N-nitroso-N-ethylurea were used. However, EMS is the most effective and a popular method of choice. Gamma irradiation was also attempted but no commercial herbicide tolerance trait has been developed yet by using this method. Direct herbicide tolerant selection from spontaneous mutation was also in use and resulted in the discovery of several herbicide tolerant mutants [10–11].

Herbicide resistant mutant plants have been identified in many crops like maize [12–13], wheat [14], cotton [15], sorghum [16], sunflower [17], lentil [18] and soybean [6]. The aim of the present study is to develop herbicide resistant plants in lentil through seed mutagenesis which can be used in herbicide resistance lentil breeding.

## Materials and methods

### Study site and experimental material

The research work was conducted at Plant Breeding and Genetics Division, Nuclear Institute for Agriculture and Biology (NIAB), Faisalabad, Pakistan during the years 2013–2016. The experimental material consisted of three advanced genotypes of lentil developed at NIAB, Faisalabad. The pedigree and genetic makeup of genotypes used in the study is given in Table 1.

**Table 1:** Description of lentil genotypes used in present study.

Sr. #	Genotype	Pedigree	Genetic makeup
1	LPP 11001	NL 20–24 × Masoor 93	Hybrid
2	LPP 11100	NL 30-5-2 × (NL 96 × Masoor 93)	Hybrid
3	LPP 11116	NL 96680/100 Gy	Mutant

### Experimental procedure

The whole experimental procedure was divided into different steps i.e. seed mutagenesis, raising of M_1_, M_2_ generations and screening against herbicides. Overall experiment detail is summarized in Table 2.

**Table 2:** Experiment detail consisting of variables, their levels and number.

Variables	Details	Number
Genotypes	LPP 11001, LPP 11100 and LPP 11116	03
Chemical mutagens	Ethyl methanesulfonate (EMS), Hydrazine Hydrate (HH) and Sodium Azide (SA),	03
Doses of each mutagen	EMS (0.1% & 0.2%), SA (0.01% & 0.02%), HH (0.02% & 0.03%)	02
Herbicides	Ally Max (Metsulfuron methyl, Tribenuron methyl) and Atlantis (Mesosulfuron methyl, Idosulfuron methyl)	02
Doses of each herbicide	Ally Max = X (34.58 g/ha), 1.5X (51.87 g/ha) Atlantis = X (395.2 g/ha), 1.5X (592.8 g/ha)	02
Screening methods	Post Plant Emergence (PPE), Pre-Plant Incorporation (PPI) and Seed Priming (SP)	03
**Treatment combinations**		**216**

### Seed mutagenesis

Healthy seeds (10 g) of each genotype were presoaked in distilled water for 16 hours [14] and then treated with two different concentrations of EMS (0.1% and 0.2%), HH (0.02% and 0.03%) and SA (0.01% and 0.02%). These mutagen doses were selected on the bases of previous reports [14, 19, 20]. Mutagen solutions were prepared in potassium phosphate buffer at pH 7.0 for 03 hours [14] with continuous intermittent shaking. The treated seeds were washed with running tap water for 5 times to remove the mutagen sticking to the seed coat. One set of seeds was kept untreated in buffer solution to serve as a comparative control.

### Raising of M_1_ and M_2_ generations

The treated seeds were dried on filter paper and four rows of each dose were planted in the field along with one row of control (untreated parent) to raise the M_1_ generation. The resulting plants were allowed to self-pollinate. The seeds of M_2_ populations were harvested from M_1_ plants and counted by using digital seed counter (Model No. 14176833). The experiment (M_2_ generation) was laid out in Augmented Split Split Plot Experiment Design (ASSPED) with three factors including screening methods as whole plot treatments, populations as split plot treatments and herbicides as split split plot treatments and “r” replicates of the whole plot treatments. The details of M_2_ populations are given in Table 3.

**Table 3:** Description of M_2_ populations of lentil derived through chemical mutagenesis.

Population #	Name/Pedigree	Total no. of seeds harvested from M_1_ Generation
1	LPP 11001/EMS 0.1%	1547
2	LPP 11001/EMS 0.2%	475
3	LPP 11001/HH 0.02%	4138
4	LPP 11001/HH 0.03%	3090
5	LPP 11001/SA 0.01%	1327
6	LPP 11001/SA 0.02%	1711
7	LPP 11116/EMS 0.1%	10769
8	LPP 11116/EMS 0.2%	3815
9	LPP 11116/HH 0.02%	9277
10	LPP 11116/HH 0.03%	10240
11	LPP 11116/SA 0.01%	9033
12	LPP 11116/SA 0.02%	6947
13	LPP 11100/EMS 0.1%	3055
14	LPP 11100/EMS 0.2%	647
15	LPP 11100/HH 0.02%	4297
16	LPP 11100/HH 0.03%	3050
17	LPP 11100/SA 0.01%	2770
18	LPP 11100/SA 0.02%	2575
19	LPP 11001/Parent	1440
20	LPP 11116/Parent	1440
21	LPP11100/Parent	1440
	**Total**	**83083**

### Screening for herbicide resistance

The M_2_ populations were screened against two different herbicides at two different doses i.e. Ally Max 28.6% SG (Metsulfuron methyl and Tribenuron methyl) at X @ 34.58 g/ha and 1.5X @ 51.87 g/ha and Atlantis 3.6% WG (Mesosulfuron methyl and Idosulfuron methyl) at X @ 395.2 g/ha and 1.5X @ 592.8 g/ha by using three different methods of herbicide application i.e. post plant emergence (PPE), pre-plant incorporation (PPI) and seed priming (SP). X is the recommended dose for both herbicides where weeds can be killed and 1.5X is the half greater than X to achieve herbicide tolerance at greater dose and to avoid the chance of weeds to become resistant to these herbicides in near future. These herbicides were used because of their broad spectrum weed control property.

In PPE method, herbicides were sprayed on plants after 80 days of emergence of the plants by using Knapsack Sprayer with Flood Jet nozzle. In PPI method, herbicides were sprayed on soil before sowing and the soil was inverted with rotary for uniform mixing of herbicide. Seeds were planted by dibbling as single seed per hill. The rows of untreated controls were covered with polythene sheet. In SP method, seeds were soaked in herbicide solution for 16 hour with frequent intermittent shaking. After that the treated seeds were washed with double distilled water, dried on filter paper and planted in field. Water untreated seeds were also sown in separate rows as control.

### Data collection

Data on following plant traits were recorded:

### Survival percentage

Herbicide resistant, tolerant and moderately tolerant plants in each treatment were considered as survived plants ignoring sensitive and highly sensitive plants. Survived plants were counted and survival percentage in each treatment was estimated by using the following formula:

$$Survival\;Percentage=\frac{Number\;of\;plants\;survived}{Total\;number\;of\;plants\;observed}\times 100$$

### Survival index

Plant survival index against herbicide treatments was calculated using the damage assessed weighted by plant number in each category and was calculated by the formula outlined by Pandolfo *et al*. (2013) [21].

$$Survival\;Index=\left(PN_{1}\times 1\right)+\left(PN_{2}\times 0.75\right)+\left(PN_{3}\times 0.5\right)+\left(PN_{4}\times 0.25\right)+\left(PN_{5}\times 0\right)/N$$

Where

*PN*_*n*_ = *plant number with n category (n = 1–5)*

*N = number of plants per* *plot*

### Selection of mutants

**Fig 1:**
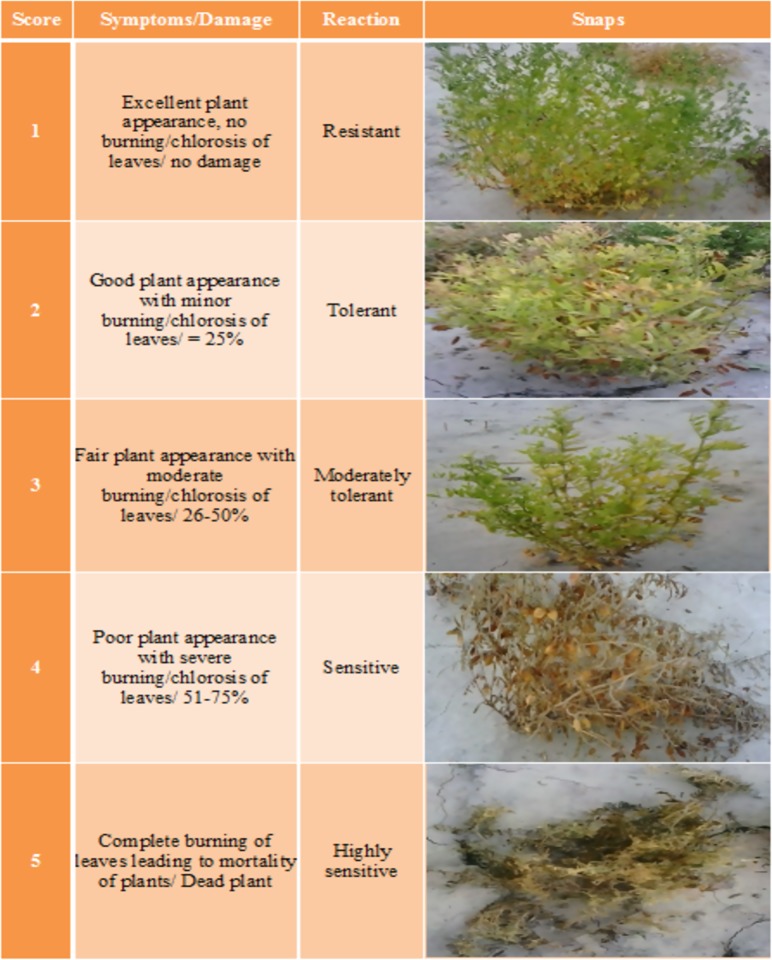
Scale used for categorizing lentil plants for their reaction against herbicides.

All plants in each treatment were categorized for their reaction against herbicides by using the modified scale given by Gaur *et al*. (2013) [22] and the herbicide resistant mutant plants were selected. The scale is given in Fig 1.

### Statistical analysis

Analysis of variance was carried out for each plant trait following Federer and Arguillas, (2005a) [23] and Federer and Arguillas, (2005b) [24] using the Statistical Analysis System SAS 9.3.1. SAS code for data analysis under ASSPED is given in S1 Table.

### Re-mutagenesis and screening at higher dose of herbicides

The seeds of selected herbicide resistant plants from the screening of M_2_ generation were re-mutagenized at 150 Gy dose of gamma radiation followed by Malkawi et al. [25]. Single plant progenies were sown in field and evaluated at 1.5X dose of respective herbicides.

## Results

The newly developed populations at M_2_ stage along with control were evaluated for different traits which can contribute to screening and selection of herbicide resistant mutants. The response of the evaluated material (S2 Table) is presented and discussed below.

### Survival index

**Fig 2:**
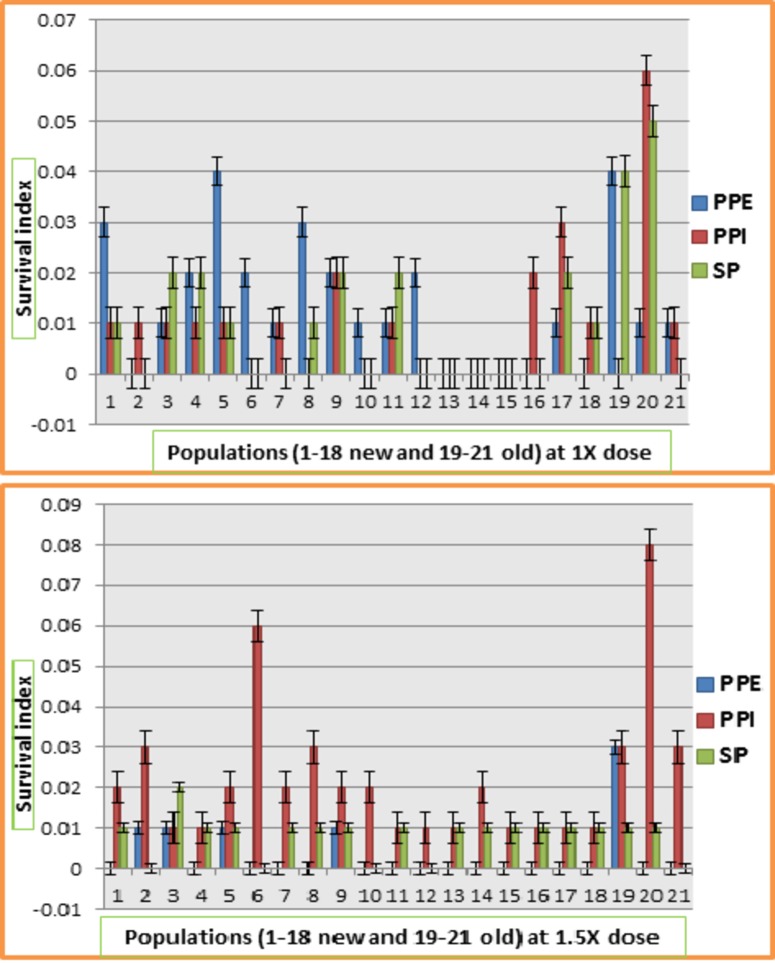
Response of 21 populations of lentil for survival index at different doses of Ally max at three different screening methods.

All populations were categorized into five categories (resistant, tolerant, moderately tolerant, sensitive and highly sensitive) according to the scale presented in Fig 1. Plant survival index was calculated using the damage assessed weighted by plant number in each category. Analysis of variance for survival index data is presented in Table 4. Highly significant differences were observed for sources of variation including model, replications, screening methods, herbicides and herbicides × screening methods interactions. Significant differences were observed for populations. Response of different populations for survival index at 1X and 1.5X dose of Ally max in all three screening methods is given in Fig 2. At 1X dose, all populations behaved differently. Populations 13, 14 and 15 showed zero survival indexes whereas population 20 showed highest survival index. Population 2 showed zero survival index in post plant emergence and seed priming screening while populations 7 and 21 showed zero survival index in seed priming method of screening. At 1.5X dose of Ally max, all populations were severely affected by PPE application of herbicide followed by SP and PPI. Population 20 showed maximum survival index followed by population 6 and population 8 in PPI screening. Populations 1, 4, 6, 7, 8, 10, 11, 12, 13, 14, 15, 16, 17, 18, 20 and 21 showed zero survival indexes in screening through PPE herbicide application.

**Table 4:** Analysis of variance for survival index and survival % of 21 populations of lentil screened through different methods against different doses of herbicides.

Source of variation	Degrees of Freedom	Mean Squares	
		Survival Index	Survival %
Model	269	0.006**	68.39**
Error	54	0.002	35.30
Corrected Total	323		
Replicate (R)	2	0.023**	323.15**
Screening methods (S)	2	0.114**	811.78**
R × S = Error S	4	0.005	65.64
Populations (P)	20	0.001**	32.00**
S × P	40	0.0006	29.34
P × R within S	12	0.002	10.55
Herbicides (H)	3	0.22**	1982.32**
H × S	6	0.094**	824.16**
H × P	60	0.001	18.01
H × S × P	120	0.001	25.14

p ≤0.01 = Highly significant**, p ≤0.05 = Significant and p >0.05 = Non-significant.

**Fig 3:**
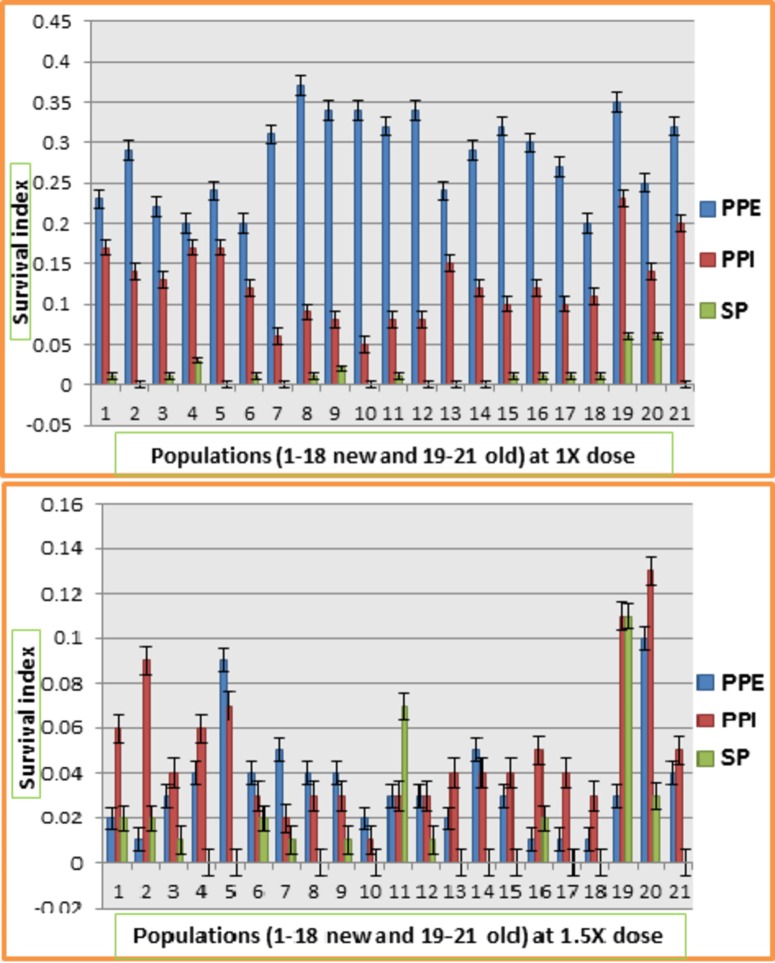
Response of 21 populations of lentil for survival index at different doses of Atlantis at three different screening methods.

Fig 3 shows the response of populations for survival index at 1X and 1.5X dose of Atlantis. In 1X environment, all populations were severely affected in SP method of screening. Highest survival index was shown by populations in PPE application followed by PPI and SP. In PPE screening, population 7 showed maximum survival index followed by populations 19 and 9. In PPI, population 19 showed maximum survival index whereas population 10 showed minimum survival index. At 1.5X dose of Atlantis herbicide, all populations were severely affected in SP screening followed by PPI and post plant emergence screening. Populations 19 and 20 showed highest survival index in all three methods of herbicide application whereas the lowest survival index was observed for population 10. Generally, it was observed that Ally max had severe effect on all population in PPE application whereas Atlantis showed severe effect on all populations screened through SP technique.

### Survival percentage

**Fig 4:**
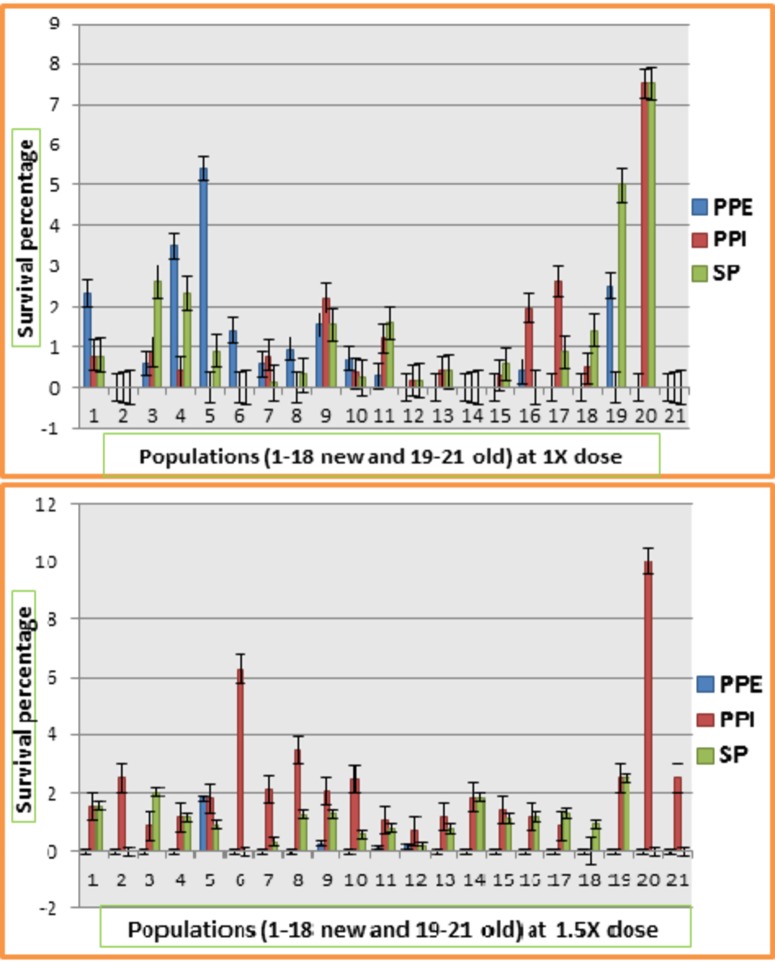
Response of 21 populations of lentil for survival percentage at different doses of Ally max at three different screening methods.

**Fig 5:**
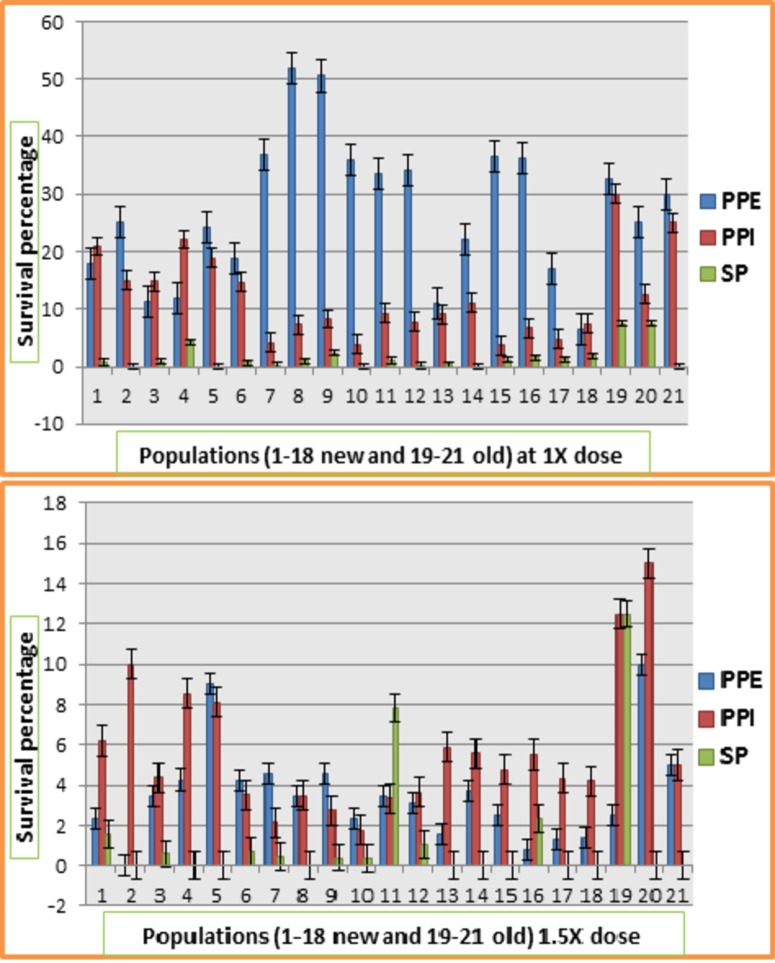
Response of 21 populations of lentil for survival percentage at different doses of Atlantis at three different screening methods.

This refers to the percentage of plants survived after herbicide treatment out of total number of plants evaluated. The analysis of variance (Table 4) showed highly significant differences among replicates, screening methods, herbicides and herbicides × screening methods interaction. Significant differences were observed among populations including model. Non-significant differences were observed for all other interactions. Response of populations for survival percentage at 1X and 1.5X dose of Ally max in all three screening methods is given in Fig 4. Survival percentage of most of the populations ranged between 0 to 3% in all environments except populations 4, 5, 19, and 20. Population 20 showed maximum survival percentage (7.5%) in both PPI and SP technique. Population 5 showed 5.41% survival in PPE application of herbicide whereas population 19 showed 5% survival in SP method of screening. At 1.5X dose of Ally max, PPE application had severe effect on survival of plants in all populations. In this environment only population 5 showed 1.8% survival. In PPI, population 20 showed maximum survival percentage of 10% followed by population 6 which had survival rate of 6.29%. In SP method of screening, population 19 showed 2.5% survival followed by population 3 which had survival rate of 2.03%. Fig 5 showed the response of populations for survival percentage at 1X and 1.5X dose of Atlantis in all screening methods. In 1X environment, survival percentage was quite high as compared to other environments. PPE application of this dose resulted in high survival rate for most of the populations including populations 8 and 9 which showed 51.9% and 50.5% survival rate respectively. PPI of this dose of herbicide also resulted in high survival percentage but less than post plant emergence application environment. 1X dose of Atlantis had shown severe effect on survival of plants in all populations in SP screening environment. Only population 19 and 20 showed 7.5% survival whereas remaining all populations showed 0 to 4.25% survival. High dose (1.5X) of this herbicide also had drastic effect on survival percentage of plants in all populations. But here one difference was observed that this high dose had also reduced the survival percentage of all populations through PPE application opposite to 1X dose of this herbicide. Population 20 showed 10% survival in PPE environment followed by population 5 which showed 9.01% survival. In PPI screening, population 20 showed maximum survival percentage of 15% followed by 10% survival of population 2. In SP method of screening, population 19 showed maximum survival rate of 12.5% followed by population 11 which had 7.84% survival. Remaining populations were found sensitive to highly sensitive in this environment.

### Selection of herbicide resistant mutants

Field reaction of screening populations is presented in S3 Table. At Ally max (1X dose) through PPE screening one resistant plant was selected from population 19 (parent) whereas population 1, 4 and 6 resulted in 1, 2 and 1 resistant mutants respectively. One resistant mutant was selected out of 897 plants studied under population 7 and 2 from population 9. Population 10 resulted in 3 resistant plants. No mutant plant was found resistant in remaining populations. At 1.5X dose of Ally max in screening through PPE method only one resistant plant was selected from population 12. At 1X dose of Atlantis in screening through PPE, 177 resistant plants were selected from all populations out of total number of 6686 plants studied. Maximum selections were made in this environment but in field conditions it was observed that these selections need further testing against higher dose of this herbicide. Although the 1X dose of Atlantis used was recommended dose in wheat crop but it was not found efficient in controlling weeds. At 1.5X dose, total number of selections made from all populations was 94 out of total plant population of 6686. Maximum resistant mutants (20) were selected from population 7. Overall, 283 resistant mutants were selected out of 26744 M_2_ plants against both herbicides in PPE screening.

In PPI screening, total 32 selections were made at 1X dose of Ally max. Maximum selections of resistant plants (08) were made in population 9 followed by 05 selections in each population 12 and 17. Most of the plants (6450) in all populations were observed to be highly sensitive. At 1.5X dose of Ally max in screening through PPI method, total 26 mutant plants were selected with maximum selections (05) from each population 6 and 7. At this dose, 6449 plants were found highly sensitive. At 1X dose of Atlantis, 159 plants were selected from all population. Population 9 showed maximum (25) resistant plants followed by 20 selections from each population 3 and 7. In total 5287 plants were observed to be highly sensitive against this dose of Atlantis. Generally, it was observed that lower dose of Atlantis in all screenings resulted in maximum number of resistant plants which needs further testing under controlled conditions at higher doses. 1.5X dose of Atlantis resulted in 70 resistant plants with maximum selections (08) made from population 11 followed by 07 selections from population 7. Number of tolerant plants was 85 in this environment whereas moderately tolerant plants were 101 in number. Total 6261 plants were observed to be highly sensitive.

In SP method of screening, total 32 selections of herbicide resistant mutants were made from 1X dose of Ally max. Maximum resistant plants (07) were selected from population 12 followed by population 2 (06 selections) and population 9 (06 selections). 13 plants were observed to be tolerant whereas 15 plants were moderately tolerant from all populations. 63 plants were sensitive whereas 6564 plants were observed to be highly sensitive. Overall, most of the plants were observed to be highly sensitive as compared to other environments of PPE and PPI screening. 1.5X dose of Ally max resulted in 19 resistant plants with maximum selections made from population 9. Only 13 plants were observed to be tolerant and 21 were moderately tolerant. 43 plants were sensitive and 6571 were highly sensitive.

**Fig 6:**
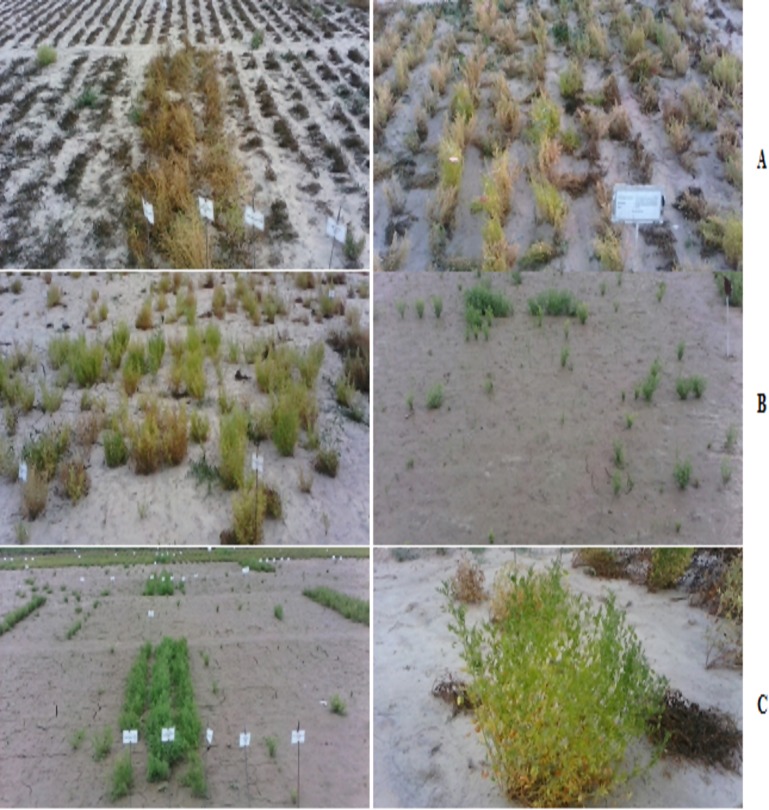
Reaction of M_2_ populations of lentil against different herbicides in screening through different methods (A = Post plant emergence, B = Pre-plant incorporation and C = Seed priming).

At 1X dose of Atlantis in screening through SP method, most of the plants (6570 plants) were observed to be highly sensitive and selections for resistant plants made were less in number (26 plants) as compared to populations screened at 1X dose in PPE and PPI screening. At 1.5X dose of Atlantis only 24 plants were selected as resistant mutants with maximum selections (19 plants) from population 11. Most of the plants (6456 plants) were observed to be highly sensitive out of total population of 6686 plants. Reaction of M_2_ populations against different herbicides in screening through different methods is shown in Fig 6.

Total six hundred and seventy one resistant plants were selected. The seeds obtained from these plants were re-mutagenized at 150 Gy dose of gamma radiation and single plant progenies were sown in field. Screening was performed against respective herbicides at 1.5X dose. One hundred and thirty four plants were survived against higher dose and designated as herbicide resistant mutants.

## Discussion

The present study showed EMS, HH and SA chemical mutagens to be efficient tools in developing herbicide resistant genotypes in lentil. These mutagens can be effectively used to generate mutations in lentil that ultimately produces resistance in plants against herbicides. Many other studies reported that induced mutations are efficient in creation of herbicide resistant mutants in different crops [14, 16, 25, 26].

In the present study, a huge number of plant populations were studied in ASSPED with three factors including screening methods as whole plot treatments, populations as split plot treatments and herbicides as split split plot treatments and “r” replicates of the whole plot treatments because of its greater flexibility to handle large number of treatments. Federer and Arguillas (2005a) [23] devised this design for such kind of experiments where large number of treatments can be studied. This study examined a large number of M_2_ population owing to the less frequency of resistant mutants to occur in a population. Kidwell *et al*. (2004) [27] reported a study in which approximately 7 million plants of wheat were screened for resistance against glyphosate.

A single wheat line Louise FRI-62 resistant to 2X dose of glyphosate was selected from continuous field and greenhouse screening. Sebastian and Chaleff (1987) [6] performed screening of M_2_ seeds from 5000 M_1_ plants against chlorsulfuron herbicide and selected seventy five putative mutants based on their ability to form true leaves within several weeks after treatment. Newhouse *et al*. (1992) [14] studied 120,000 M_2_ plants for the development of imidazolinone resistant wheat. Ndungu (2009) [16] used 0.3% EMS to mutagenize 50,000 seeds and screened over four million M_2_ plants against sulfosulfuron. Generally, it is not easy to develop herbicide resistance in crop plants at higher doses of herbicides which can kill all types of weeds in a single application. But new strategies like large scale screening of mutagenized populations, re-mutagenesis and screening and gene pyramiding of independently isolated mutations are being adopted to increase the resistance of crop plants against higher doses of herbicides. In the present study, different screening methods were used like PPE, PPI and SP for screening of mutant populations against herbicides. Some other workers used these screening techniques and selected putative herbicide resistant mutants in different crops [6, 14].

From M_2_ screening, the present study selected 671 resistant plants (121 resistant to Ally max and 550 to Atlantis) based on their excellent appearance/no burning and chlorosis of leaves after application of herbicides. Under field conditions, symptoms were clear and most of the plants from studied population were found highly sensitive to the applied herbicides. Out of 671 resistant plants, 437 were resistant at lower dose (1X) of both herbicides whereas 234 were resistant at high dose (1.5X). Earlier, many researchers worked on developing herbicide resistant crops through chemical mutagenesis. Chant (2004) [26] developed an imidazolinone tolerant lentil mutant (RH44) by EMS mutagenesis. Malkawi *et al*., (2003) [25] treated lentil cultivars with gamma radiations to develop tolerance against chlorsulfuron herbicide. Similarly, Sebastian and Chaleff (1987) [6] selected four mutants of soybean (1-126A, 1-166A, 1-183A and 1-184A) which appeared to be sufficiently tolerant to chlorsulfuron herbicide. Newhouse *et al*. (1992) [14] also selected four lines of wheat (FS1, FS2, FS3 and FS4) that were confirmed to be imidazolinone resistant. Ndungu (2009) [16] developed acetolactate synthase herbicide resistance in sorghum. The author selected five mutants’ hb46, hb462, hb56, hb8 and hb12 resistant against sulfosulfuron. These mutants showed differential herbicide tolerance and their general order of tolerance after seed coat application was hb46>hb12>hb462>hb56>hb8.

In the present study, the selected resistant plants were re-mutagenized and evaluated against 1.5X dose of respective herbicides. Out of 671 single plant progenies evaluated in field conditions, only 134 resistant plants (42 Ally max and 92 Atlantis resistant) were selected. The remaining plant population showed moderately tolerant to highly sensitive response to the applied herbicides. Kidwell *et al*. (2004) [27] also used re-mutagenesis technique to increase the level of resistance from 2X to 4X in glyphosate resistant wheat mutants followed by gene pyramiding of independently isolated mutations. The finally selected herbicide resistant mutants from this study can be advanced further through screening under controlled conditions. Moreover, biochemical (acetolactate synthase assay) and molecular studies can be accomplished for the genetic confirmation of true herbicide resistant mutants.

## Conclusion

A total of 83083 plants in 21 populations were screened using 12 different testing environments of herbicides, doses and screening methods. A total 671 plants were selected as resistant plants. The seeds from these plants after re-mutagenesis were again evaluated against higher dose of respective herbicides. Overall 134 herbicide resistant plants (42 Ally max and 92 Atlantis resistant) were selected. The selected mutants are useful germplasm for herbicide resistance breeding of lentil.

## Supporting information

S1 Table SAS code for data analysis under ASSPED (Example S1 Table survival percentage response). (DOCX)

S2 Table Responses for the ASSPED. (DOCX)

S3 Table Field reaction of M_2_ populations against herbicides. (DOCX)
