# Supplementary material for: Pre-breeding of lentil (Lens culinaris Medik.) for herbicide resistance through seed mutagenesis
Source: PLoS One. 2017 Feb 14;12(2):e0171846. doi: 10.1371/journal.pone.0171846 (PMC5308809; doi:10.1371/journal.pone.0171846)
Supplement: S1 Table — (DOCX) [file pone.0171846.s001.docx]

**S1 Table. SAS code for data analysis under ASSPED (Example S1 Table Survival percentage response)**

The code for example Appendix-A is given below. The least squares means for other than checks and interactions of S and H with checks are non-estimable.

data assped;

input Y R S P H;

if (P > 20) then new = 0; else new = 1;

if (new) then Pn = 999; else Pn = P;

datalines;

| 2.33 | 1 | 1 | 1 | 1 |
| --- | --- | --- | --- | --- |
| 0.00 | 1 | 1 | 1 | 2 |
| 17.8 | 1 | 1 | 1 | 3 |
| 2.33 | 1 | 1 | 1 | 4 |
| 0.00 | 1 | 1 | 2 | 1 |
| 0.00 | 1 | 1 | 2 | 2 |
| 25.0 | 1 | 1 | 2 | 3 |
| 0.00 | 1 | 1 | 2 | 4 |
|  |  |  |  |  |
| …….. |  |  |  |  |
|  |  |  |  |  |
| 3.50 | 3 | 3 | 20 | 1 |
| 6.50 | 3 | 3 | 20 | 2 |
| 0.00 | 3 | 3 | 20 | 3 |
| 0.00 | 3 | 3 | 20 | 4 |
| 1.50 | 3 | 3 | 21 | 1 |
| 0.00 | 3 | 3 | 21 | 2 |
| 0.00 | 3 | 3 | 21 | 3 |
| 5.00 | 3 | 3 | 21 | 4 |

;

ODS RTF FILE="C:\Users\Acer\Desktop\OUTPUT.DOC";

PROC PRINT DATA=ASSPED;

RUN;

ods trace on;

proc glm data = ASSPED;

class R S P H;

model Y=R S R*S P P*S R*P(S) H H*S H*P H*P*S/solution;

/*only means estimable are check P means*/;

lsmeans S P S*P H H*S H*P H*P*S/out=solution;

ods output parameterestimates=asspedparameterestimates;

ods trace off;

RUN;

proc sort data=asspedparameterestimates;

by descending estimates;

RUN;

proc print DATA=asspedparameterestimates;

run;

ods listing close; /*Unordered solutions not printed.*/

ods trace on;

proc mixed data=assped;

class R S P H Pn;

model Y=S Pn Pn*S H H*S H*Pn H*Pn*S/solution;

random R R*S Pn*R(S) P*new/solution;

lsmeans S Pn Pn*S H H*Pn H*S H*Pn*S;

ods output solutionf=solutionforfixedeffects;

ods output solutionr=solutionforrandomeffects;

ods output lsmeans=leastsquaremeans;

run;

ods trace off;

ods listing; /*End of no printing statement.*/

RUN;

proc sort data=solutionforfixedeffects;

by descending estimates;

RUN;

proc print DATA=solutionforfixedeffects;

run;

proc sort data=solutionforrandomeffects;

by descending estimate;

RUN;

proc print DATA=solutionforrandomeffects;

run;

proc sort data=leastsquaremeans;

by descending estimate;

RUN;

proc print DATA=leastsquaremeans;

run;

ODS RTF CLOSE;
