# Supplementary material for: Pre-breeding of lentil (Lens culinaris Medik.) for herbicide resistance through seed mutagenesis
Source: PLoS One. 2017 Feb 14;12(2):e0171846. doi: 10.1371/journal.pone.0171846 (PMC5308809; doi:10.1371/journal.pone.0171846)
Supplement: S2 Table — (DOCX) [file pone.0171846.s002.docx]

**S2 Table. Responses for the ASSPED**

**Survival index responses of different populations as split plot, herbicides as split split plot and screening methods as whole plot treatment for the ASSPED**

| **R1** |  |  |  |  |  |  |  |  |  |  |  |  |  |  |  |  |  |  |  |  |  |  |  |  |  |  |  |  |  |
| --- | --- | --- | --- | --- | --- | --- | --- | --- | --- | --- | --- | --- | --- | --- | --- | --- | --- | --- | --- | --- | --- | --- | --- | --- | --- | --- | --- | --- | --- |
|  | **S1** | | | |  |  |  |  |  |  | **S2** | | | |  |  |  |  |  |  | **S3** | | | |  |  |  |  |  |
|  | **Genotype** | | | | | | | | |  | **Genotype** | | | | | | | | |  | **Genotype** | | | | | | | | |
| **H** | **1** | **2** | **3** | **4** | **5** | **6** | **19** | **20** | **21** | **H** | **1** | **2** | **3** | **4** | **5** | **6** | **19** | **20** | **21** | **H** | **1** | **2** | **3** | **4** | **5** | **6** | **19** | **20** | **21** |
| H1X1 | 0.03 | 0.00 | 0.01 | 0.02 | 0.04 | 0.02 | 0.04 | 0.01 | 0.01 | H1X1 | 0.01 | 0.01 | 0.01 | 0.01 | 0.01 | 0.00 | 0.00 | 0.06 | 0.01 | H1X1 | 0.01 | 0.00 | 0.02 | 0.02 | 0.01 | 0.00 | 0.04 | 0.05 | 0.00 |
| H1X2 | 0.00 | 0.01 | 0.01 | 0.00 | 0.01 | 0.00 | 0.03 | 0.00 | 0.00 | H1X2 | 0.02 | 0.03 | 0.01 | 0.01 | 0.02 | 0.06 | 0.03 | 0.08 | 0.03 | H1X2 | 0.01 | 0.00 | 0.02 | 0.01 | 0.01 | 0.00 | 0.01 | 0.01 | 0.00 |
| H2X1 | 0.23 | 0.29 | 0.22 | 0.20 | 0.24 | 0.20 | 0.35 | 0.25 | 0.32 | H2X1 | 0.17 | 0.14 | 0.13 | 0.17 | 0.17 | 0.12 | 0.23 | 0.14 | 0.20 | H2X1 | 0.01 | 0.00 | 0.01 | 0.03 | 0.00 | 0.01 | 0.06 | 0.06 | 0.00 |
| H2X2 | 0.02 | 0.01 | 0.03 | 0.04 | 0.09 | 0.04 | 0.03 | 0.1 | 0.04 | H2X2 | 0.06 | 0.09 | 0.04 | 0.06 | 0.07 | 0.03 | 0.11 | 0.13 | 0.05 | H2X2 | 0.02 | 0.02 | 0.01 | 0.00 | 0.00 | 0.02 | 0.11 | 0.03 | 0.00 |
| **R2** |  |  |  |  |  |  |  |  |  |  |  |  |  |  |  |  |  |  |  |  |  |  |  |  |  |  |  |  |  |
|  | **S1** | | | |  |  |  |  |  |  | **S2** | | | |  |  |  |  |  |  | **S3** | | | |  |  |  |  |  |
|  | **Genotype** | | | | | | | | |  | **Genotype** | | | | | | | | |  | **Genotype** | | | | | | | | |
| **H** | **7** | **8** | **9** | **10** | **11** | **12** | **19** | **20** | **21** | **H** | **7** | **8** | **9** | **10** | **11** | **12** | **19** | **20** | **21** | **H** | **7** | **8** | **9** | **10** | **11** | **12** | **19** | **20** | **21** |
| H1X1 | 0.01 | 0.03 | 0.02 | 0.01 | 0.01 | 0.02 | 0.00 | 0.03 | 0.02 | H1X1 | 0.01 | 0.00 | 0.02 | 0.00 | 0.01 | 0.00 | 0.01 | 0.01 | 0.01 | H1X1 | 0.00 | 0.01 | 0.02 | 0.00 | 0.02 | 0.00 | 0.01 | 0.04 | 0.00 |
| H1X2 | 0.00 | 0.00 | 0.01 | 0.00 | 0.00 | 0.00 | 0.00 | 0.00 | 0.01 | H1X2 | 0.02 | 0.03 | 0.02 | 0.02 | 0.01 | 0.01 | 0.02 | 0.01 | 0.00 | H1X2 | 0.01 | 0.01 | 0.01 | 0.00 | 0.01 | 0.00 | 0.00 | 0.02 | 0.06 |
| H2X1 | 0.31 | 0.37 | 0.34 | 0.34 | 0.32 | 0.34 | 0.21 | 0.06 | 0.25 | H2X1 | 0.06 | 0.09 | 0.08 | 0.05 | 0.08 | 0.08 | 0.00 | 0.06 | 0.04 | H2X1 | 0.00 | 0.01 | 0.02 | 0.00 | 0.01 | 0.00 | 0.02 | 0.00 | 0.00 |
| H2X2 | 0.05 | 0.04 | 0.04 | 0.02 | 0.03 | 0.03 | 0.00 | 0.36 | 0.00 | H2X2 | 0.02 | 0.03 | 0.03 | 0.01 | 0.03 | 0.03 | 0.04 | 0.00 | 0.01 | H2X2 | 0.01 | 0.00 | 0.01 | 0.00 | 0.07 | 0.01 | 0.01 | 0.00 | 0.08 |
| **R3** |  |  |  |  |  |  |  |  |  |  |  |  |  |  |  |  |  |  |  |  |  |  |  |  |  |  |  |  |  |
|  | **S1** | | | |  |  |  |  |  |  | **S2** | | | |  |  |  |  |  |  | **S3** | | | |  |  |  |  |  |
|  | **Genotype** | | | | | | | | |  | **Genotype** | | | | | | | | |  | **Genotype** | | | | | | | | |
| **H** | **13** | **14** | **15** | **16** | **17** | **18** | **19** | **20** | **21** | **H** | **13** | **14** | **15** | **16** | **17** | **18** | **19** | **20** | **21** | **H** | **13** | **14** | **15** | **16** | **17** | **18** | **19** | **20** | **21** |
| H1X1 | 0.00 | 0.00 | 0.00 | 0.00 | 0.01 | 0.00 | 0.01 | 0.00 | 0.01 | H1X1 | 0.00 | 0.00 | 0.00 | 0.02 | 0.03 | 0.01 | 0.02 | 0.04 | 0.01 | H1X1 | 0.00 | 0.00 | 0.00 | 0.00 | 0.02 | 0.01 | 0.00 | 0.03 | 0.00 |
| H1X2 | 0.00 | 0.00 | 0.00 | 0.00 | 0.00 | 0.00 | 0.00 | 0.00 | 0.01 | H1X2 | 0.01 | 0.02 | 0.01 | 0.01 | 0.01 | 0.01 | 0.00 | 0.06 | 0.00 | H1X2 | 0.01 | 0.01 | 0.01 | 0.01 | 0.01 | 0.01 | 0.02 | 0.00 | 0.03 |
| H2X1 | 0.24 | 0.29 | 0.32 | 0.3 | 0.27 | 0.20 | 0.04 | 0.01 | 0.21 | H2X1 | 0.15 | 0.12 | 0.10 | 0.12 | 0.10 | 0.11 | 0.08 | 0.03 | 0.08 | H2X1 | 0.00 | 0.00 | 0.01 | 0.01 | 0.01 | 0.01 | 0.01 | 0.04 | 0.01 |
| H2X2 | 0.02 | 0.05 | 0.03 | 0.01 | 0.01 | 0.01 | 0.03 | 0.02 | 0.01 | H2X2 | 0.04 | 0.04 | 0.04 | 0.05 | 0.04 | 0.03 | 0.02 | 0.01 | 0.00 | H2X2 | 0.00 | 0.00 | 0.00 | 0.02 | 0.00 | 0.00 | 0.00 | 0.00 | 0.00 |

R= Replications, S1= Post Plant Emergence, S2= Pre-Plant Incorporation, S3= Seed Priming, Genotypes 1-18 = New populations, Genotype 19-21 = Old Populations H= Herbicide, X= Herbicide dose, H1X1= Ally max X, H1X2= Ally max 1.5X, H2X1= Atlantis X and H2X2= Atlantis 1.5X

ASSPED= Augmented Split Split Plot Experiment Design

**Survival percentage responses of different populations as split plot, herbicides as split split plot and screening methods as whole plot treatment for ASSPED**

| **R1** |  |  |  |  |  |  |  |  |  |  |  |  |  |  |  |  |  |  |  |  |  |  |  |  |  |  |  |  |  |
| --- | --- | --- | --- | --- | --- | --- | --- | --- | --- | --- | --- | --- | --- | --- | --- | --- | --- | --- | --- | --- | --- | --- | --- | --- | --- | --- | --- | --- | --- |
|  | **S1** | | | |  |  |  |  |  |  | **S2** | | | |  |  |  |  |  |  | **S3** | | | |  |  |  |  |  |
|  | **Genotype** | | | | | | | | |  | **Genotype** | | | | | | | | |  | **Genotype** | | | | | | | | |
| **H** | **1** | **2** | **3** | **4** | **5** | **6** | **19** | **20** | **21** | **H** | **1** | **2** | **3** | **4** | **5** | **6** | **19** | **20** | **21** | **H** | **1** | **2** | **3** | **4** | **5** | **6** | **19** | **20** | **21** |
| H1X1 | 2.33 | 0.00 | 0.58 | 3.49 | 5.41 | 1.40 | 2.50 | 0.00 | 0.00 | H1X1 | 0.78 | 0.00 | 0.87 | 0.39 | 0.00 | 0.00 | 0.00 | 7.50 | 0.00 | H1X1 | 0.78 | 0.00 | 2.61 | 2.33 | 0.90 | 0.00 | 5.00 | 7.50 | 0.00 |
| H1X2 | 0.00 | 0.00 | 0.00 | 0.00 | 1.80 | 0.00 | 0.00 | 0.00 | 0.00 | H1X2 | 1.55 | 2.50 | 0.87 | 1.16 | 1.80 | 6.29 | 2.50 | 10.0 | 2.50 | H1X2 | 1.55 | 0.00 | 2.03 | 1.16 | 0.90 | 0.00 | 2.50 | 0.00 | 0.00 |
| H2X1 | 17.8 | 25.0 | 11.3 | 12.0 | 24.3 | 18.9 | 32.5 | 25.0 | 30.0 | H2X1 | 20.9 | 15.0 | 14.8 | 22.1 | 18.9 | 14.7 | 30.0 | 12.5 | 25.0 | H2X1 | 0.78 | 0.00 | 0.87 | 4.26 | 0.00 | 0.70 | 7.50 | 7.50 | 0.00 |
| H2X2 | 2.33 | 0.00 | 3.48 | 4.26 | 9.01 | 4.20 | 2.50 | 10.0 | 5.00 | H2X2 | 6.20 | 10.0 | 4.35 | 8.53 | 8.11 | 3.50 | 12.5 | 15.0 | 5.00 | H2X2 | 1.55 | 0.00 | 0.58 | 0.00 | 0.00 | 0.70 | 12.5 | 0.00 | 0.00 |
| **R2** |  |  |  |  |  |  |  |  |  |  |  |  |  |  |  |  |  |  |  |  |  |  |  |  |  |  |  |  |  |
|  | **S1** | | | |  |  |  |  |  |  | **S2** | | | |  |  |  |  |  |  | **S3** | | | |  |  |  |  |  |
|  | **Genotype** | | | | | | | | |  | **Genotype** | | | | | | | | |  | **Genotype** | | | | | | | | |
| **H** | **7** | **8** | **9** | **10** | **11** | **12** | **19** | **20** | **21** | **H** | **7** | **8** | **9** | **10** | **11** | **12** | **19** | **20** | **21** | **H** | **7** | **8** | **9** | **10** | **11** | **12** | **19** | **20** | **21** |
| H1X1 | 0.56 | 0.94 | 1.55 | 0.70 | 0.27 | 0.00 | 0.00 | 0.00 | 1.50 | H1X1 | 0.78 | 0.00 | 2.20 | 0.35 | 1.20 | 0.17 | 0.00 | 2.50 | 0.00 | H1X1 | 0.11 | 0.31 | 1.55 | 0.23 | 1.59 | 0.17 | 2.50 | 9.00 | 0.00 |
| H1X2 | 0.00 | 0.00 | 0.26 | 0.00 | 0.13 | 0.17 | 0.00 | 0.00 | 0.00 | H1X2 | 2.12 | 3.46 | 2.07 | 2.46 | 1.06 | 0.69 | 7.50 | 0.00 | 3.50 | H1X2 | 0.33 | 1.26 | 1.29 | 0.59 | 0.80 | 0.17 | 0.00 | 2.50 | 4.50 |
| H2X1 | 36.8 | 51.9 | 50.5 | 35.9 | 33.6 | 34.0 | 18.5 | 0.00 | 0.00 | H2X1 | 4.12 | 7.23 | 8.28 | 3.87 | 9.30 | 7.77 | 5.50 | 0.00 | 3.50 | H2X1 | 0.22 | 0.94 | 2.33 | 0.00 | 1.06 | 0.17 | 11.5 | 5.00 | 1.50 |
| H2X2 | 4.57 | 3.46 | 4.53 | 2.34 | 3.45 | 3.11 | 2.00 | 0.00 | 0.00 | H2X2 | 2.12 | 3.46 | 2.72 | 1.76 | 3.32 | 3.63 | 0.00 | 2.50 | 8.50 | H2X2 | 0.45 | 0.00 | 0.39 | 0.35 | 7.84 | 1.04 | 10.5 | 0.00 | 0.00 |
| **R3** |  |  |  |  |  |  |  |  |  |  |  |  |  |  |  |  |  |  |  |  |  |  |  |  |  |  |  |  |  |
|  | **S1** | | | |  |  |  |  |  |  | **S2** | | | |  |  |  |  |  |  | **S3** | | | |  |  |  |  |  |
|  | **Genotype** | | | | | | | | |  | **Genotype** | | | | | | | | |  | **Genotype** | | | | | | | | |
| **H** | **13** | **14** | **15** | **16** | **17** | **18** | **19** | **20** | **21** | **H** | **13** | **14** | **15** | **16** | **17** | **18** | **19** | **20** | **21** | **H** | **13** | **14** | **15** | **16** | **17** | **18** | **19** | **20** | **21** |
| H1X1 | 0.00 | 0.00 | 0.00 | 0.39 | 0.00 | 0.00 | 0.00 | 0.00 | 0.00 | H1X1 | 0.39 | 0.00 | 0.28 | 1.97 | 2.60 | 0.47 | 9.50 | 0.00 | 7.50 | H1X1 | 0.39 | 0.00 | 0.56 | 0.00 | 0.87 | 1.40 | 0.00 | 3.50 | 1.50 |
| H1X2 | 0.00 | 0.00 | 0.00 | 0.00 | 0.00 | 0.00 | 0.00 | 0.00 | 0.00 | H1X2 | 1.18 | 1.85 | 1.40 | 1.18 | 0.87 | 0.00 | 0.00 | 0.00 | 3.50 | H1X2 | 0.78 | 1.85 | 1.12 | 1.18 | 1.30 | 0.93 | 0.00 | 6.50 | 0.00 |
| H2X1 | 11.0 | 22.2 | 36.6 | 36.2 | 16.9 | 6.51 | 5.00 | 0.00 | 2.50 | H2X1 | 9.02 | 11.1 | 3.63 | 6.69 | 4.76 | 7.44 | 4.5 | 10.0 | 2.50 | H2X1 | 0.39 | 0.00 | 1.12 | 1.57 | 1.30 | 1.86 | 2.50 | 0.00 | 0.00 |
| H2X2 | 1.57 | 3.70 | 2.51 | 0.79 | 1.30 | 1.40 | 1.50 | 0.00 | 0.00 | H2X2 | 5.88 | 5.56 | 4.75 | 5.51 | 4.33 | 4.19 | 0.00 | 5.50 | 0.00 | H2X2 | 0.00 | 0.00 | 0.00 | 2.36 | 0.00 | 0.00 | 1.50 | 0.00 | 5.00 |

R= Replications, S1= Post Plant Emergence, S2= Pre-Plant Incorporation, S3= Seed Priming, Genotypes 1-18 = New populations, Genotype 19-21 = Old Populations H= Herbicide, X= Herbicide dose, H1X1= Ally max X, H1X2= Ally max 1.5X, H2X1= Atlantis X and H2X2= Atlantis 1.5X

ASSPED= Augmented Split Split Plot Experiment Design
