# Supplementary material for: Pre-breeding of lentil (Lens culinaris Medik.) for herbicide resistance through seed mutagenesis
Source: PLoS One. 2017 Feb 14;12(2):e0171846. doi: 10.1371/journal.pone.0171846 (PMC5308809; doi:10.1371/journal.pone.0171846)
Supplement: S3 Table — (DOCX) [file pone.0171846.s003.docx]

**S3 Table. Field Reaction of M2 Populations against herbicides**

Table 1. Field reaction of M_2_ populations to ALLY MAX (X dose) in screening through post plant emergence (PPE) method

| Genotype | Treatment | No. of plants studied | No. of plants with scoring of | | | | |
| --- | --- | --- | --- | --- | --- | --- | --- |
|  |  |  | 1 (R) | 2 (T) | 3 (MT) | 4 (S) | 5 (HS) |
| LPP 11001 | Parent | 40 | 1 | 0 | 0 | 3 | 36 |
|  | 0.1% EMS | 129 | 1 | 1 | 1 | 4 | 122 |
|  | 0.2% EMS | 40 | 0 | 0 | 0 | 0 | 40 |
|  | 0.02% HH | 345 | 0 | 0 | 2 | 4 | 339 |
|  | 0.03% HH | 258 | 2 | 0 | 7 | 3 | 246 |
|  | 0.01% SA | 111 | 0 | 1 | 5 | 3 | 102 |
|  | 0.02% SA | 143 | 1 | 0 | 1 | 4 | 137 |
| LPP 11116 | Parent | 40 | 0 | 0 | 0 | 1 | 39 |
|  | 0.1% EMS | 897 | 1 | 1 | 3 | 25 | 867 |
|  | 0.2% EMS | 318 | 0 | 0 | 3 | 32 | 283 |
|  | 0.02% HH | 773 | 2 | 2 | 8 | 35 | 726 |
|  | 0.03% HH | 853 | 3 | 0 | 3 | 9 | 838 |
|  | 0.01% SA | 753 | 0 | 0 | 2 | 17 | 734 |
|  | 0.02% SA | 579 | 0 | 0 | 0 | 35 | 544 |
| LPP 11100 | Parent | 40 | 0 | 0 | 0 | 1 | 39 |
|  | 0.1% EMS | 255 | 0 | 0 | 0 | 1 | 254 |
|  | 0.2% EMS | 54 | 0 | 0 | 0 | 0 | 54 |
|  | 0.02% HH | 358 | 0 | 0 | 0 | 3 | 355 |
|  | 0.03% HH | 254 | 0 | 0 | 1 | 2 | 251 |
|  | 0.01% SA | 231 | 0 | 0 | 0 | 5 | 226 |
|  | 0.02% SA | 215 | 0 | 0 | 0 | 4 | 211 |

^$^R = Resistant, T = Tolerant, MT = Moderately Tolerant, S = Sensitive and HS = Highly Sensitive

Table 2. Field reaction of M_2_ populations to ALLY MAX (1.5X dose) in screening through post plant emergence (PPE) method

| Genotype | Treatment | No. of plants studied | No. of plants with scoring of | | | | |
| --- | --- | --- | --- | --- | --- | --- | --- |
|  |  |  | 1 (R) | 2 (T) | 3 (MT) | 4 (S) | 5 (HS) |
| LPP 11001 | Parent | 40 | 0 | 0 | 0 | 4 | 36 |
|  | 0.1% EMS | 129 | 0 | 0 | 0 | 2 | 127 |
|  | 0.2% EMS | 40 | 0 | 0 | 0 | 1 | 39 |
|  | 0.02% HH | 345 | 0 | 0 | 0 | 8 | 337 |
|  | 0.03% HH | 258 | 0 | 0 | 0 | 3 | 255 |
|  | 0.01% SA | 111 | 0 | 0 | 2 | 0 | 109 |
|  | 0.02% SA | 143 | 0 | 0 | 0 | 0 | 143 |
| LPP 11116 | Parent | 40 | 0 | 0 | 0 | 0 | 40 |
|  | 0.1% EMS | 897 | 0 | 0 | 0 | 2 | 895 |
|  | 0.2% EMS | 318 | 0 | 0 | 0 | 2 | 316 |
|  | 0.02% HH | 773 | 0 | 0 | 2 | 13 | 758 |
|  | 0.03% HH | 853 | 0 | 0 | 0 | 6 | 846 |
|  | 0.01% SA | 753 | 0 | 0 | 1 | 2 | 750 |
|  | 0.02% SA | 579 | 1 | 0 | 0 | 2 | 576 |
| LPP 11100 | Parent | 40 | 0 | 0 | 0 | 0 | 40 |
|  | 0.1% EMS | 255 | 0 | 0 | 0 | 0 | 255 |
|  | 0.2% EMS | 54 | 0 | 0 | 0 | 0 | 54 |
|  | 0.02% HH | 358 | 0 | 0 | 0 | 1 | 357 |
|  | 0.03% HH | 254 | 0 | 0 | 0 | 0 | 254 |
|  | 0.01% SA | 231 | 0 | 0 | 0 | 0 | 231 |
|  | 0.02% SA | 215 | 0 | 0 | 0 | 0 | 215 |

^$^R = Resistant, T = Tolerant, MT = Moderately Tolerant, S = Sensitive and HS = Highly Sensitive

Table 3. Field reaction of M_2_ populations to ATLANTIS (X dose) in screening through post plant emergence (PPE) method

| Genotype | Treatment | No. of plants studied | No. of plants with scoring of | | | | |
| --- | --- | --- | --- | --- | --- | --- | --- |
|  |  |  | 1 (R) | 2 (T) | 3 (MT) | 4 (S) | 5 (HS) |
| LPP 11001 | Parent | 40 | 3 | 4 | 6 | 20 | 7 |
|  | 0.1% EMS | 129 | 6 | 7 | 10 | 53 | 53 |
|  | 0.2% EMS | 40 | 2 | 3 | 5 | 20 | 10 |
|  | 0.02% HH | 345 | 7 | 15 | 17 | 201 | 105 |
|  | 0.03% HH | 258 | 5 | 11 | 15 | 125 | 102 |
|  | 0.01% SA | 111 | 1 | 12 | 14 | 39 | 45 |
|  | 0.02% SA | 143 | 3 | 9 | 15 | 43 | 73 |
| LPP 11116 | Parent | 40 | 4 | 1 | 5 | 11 | 19 |
|  | 0.1% EMS | 897 | 24 | 105 | 201 | 293 | 274 |
|  | 0.2% EMS | 318 | 5 | 59 | 101 | 68 | 85 |
|  | 0.02% HH | 773 | 12 | 117 | 261 | 130 | 253 |
|  | 0.03% HH | 853 | 18 | 107 | 181 | 401 | 146 |
|  | 0.01% SA | 753 | 20 | 89 | 144 | 330 | 170 |
|  | 0.02% SA | 579 | 27 | 67 | 103 | 270 | 112 |
| LPP 11100 | Parent | 40 | 3 | 4 | 5 | 17 | 11 |
|  | 0.1% EMS | 255 | 10 | 7 | 11 | 165 | 62 |
|  | 0.2% EMS | 54 | 2 | 4 | 6 | 31 | 11 |
|  | 0.02% HH | 358 | 13 | 21 | 97 | 146 | 81 |
|  | 0.03% HH | 254 | 6 | 13 | 73 | 96 | 66 |
|  | 0.01% SA | 231 | 5 | 11 | 23 | 151 | 41 |
|  | 0.02% SA | 215 | 1 | 4 | 9 | 138 | 63 |

^$^R = Resistant, T = Tolerant, MT = Moderately Tolerant, S = Sensitive and HS = Highly Sensitive

Table 4. Field reaction of M_2_ populations to ATLANTIS (1.5X dose) in screening through post plant emergence (PPE) method

| Genotype | Treatment | No. of plants studied | No. of plants with scoring of | | | | |
| --- | --- | --- | --- | --- | --- | --- | --- |
|  |  |  | 1 (R) | 2 (T) | 3 (MT) | 4 (S) | 5 (HS) |
| LPP 11001 | Parent | 40 | 0 | 0 | 1 | 3 | 36 |
|  | 0.1% EMS | 129 | 1 | 1 | 1 | 3 | 112 |
|  | 0.2% EMS | 40 | 0 | 0 | 0 | 2 | 38 |
|  | 0.02% HH | 345 | 6 | 1 | 5 | 9 | 324 |
|  | 0.03% HH | 258 | 7 | 1 | 3 | 4 | 243 |
|  | 0.01% SA | 111 | 7 | 0 | 3 | 6 | 95 |
|  | 0.02% SA | 143 | 4 | 0 | 2 | 4 | 133 |
| LPP 11116 | Parent | 40 | 1 | 1 | 2 | 5 | 31 |
|  | 0.1% EMS | 897 | 20 | 14 | 7 | 29 | 827 |
|  | 0.2% EMS | 318 | 6 | 3 | 2 | 11 | 296 |
|  | 0.02% HH | 773 | 10 | 16 | 9 | 21 | 717 |
|  | 0.03% HH | 853 | 7 | 2 | 11 | 24 | 809 |
|  | 0.01% SA | 753 | 12 | 5 | 9 | 18 | 709 |
|  | 0.02% SA | 579 | 7 | 4 | 7 | 17 | 544 |
| LPP 11100 | Parent | 40 | 0 | 0 | 2 | 3 | 35 |
|  | 0.1% EMS | 255 | 1 | 0 | 3 | 6 | 245 |
|  | 0.2% EMS | 54 | 1 | 1 | 0 | 3 | 50 |
|  | 0.02% HH | 358 | 2 | 3 | 4 | 14 | 335 |
|  | 0.03% HH | 254 | 1 | 0 | 1 | 9 | 243 |
|  | 0.01% SA | 231 | 1 | 0 | 2 | 4 | 224 |
|  | 0.02% SA | 215 | 0 | 1 | 2 | 3 | 209 |

^$^R = Resistant, T = Tolerant, MT = Moderately Tolerant, S = Sensitive and HS = Highly Sensitive

Table 5. Field reaction of M_2_ populations to ALLY MAX (X dose) in screening through pre plant incorporation (PPI) method

| Genotype | Treatment | No. of plants studied | No. of plants with scoring of | | | | |
| --- | --- | --- | --- | --- | --- | --- | --- |
|  |  |  | 1 (R) | 2 (T) | 3 (MT) | 4 (S) | 5 (HS) |
| LPP 11001 | Parent | 40 | 0 | 0 | 0 | 0 | 40 |
|  | 0.1% EMS | 129 | 1 | 0 | 0 | 0 | 128 |
|  | 0.2% EMS | 40 | 0 | 0 | 0 | 2 | 38 |
|  | 0.02% HH | 345 | 1 | 2 | 0 | 3 | 339 |
|  | 0.03% HH | 258 | 0 | 0 | 1 | 8 | 249 |
|  | 0.01% SA | 111 | 0 | 0 | 0 | 3 | 108 |
|  | 0.02% SA | 143 | 0 | 0 | 0 | 1 | 142 |
| LPP 11116 | Parent | 40 | 1 | 0 | 2 | 2 | 35 |
|  | 0.1% EMS | 897 | 3 | 1 | 3 | 8 | 882 |
|  | 0.2% EMS | 318 | 0 | 0 | 0 | 2 | 316 |
|  | 0.02% HH | 773 | 8 | 3 | 6 | 8 | 748 |
|  | 0.03% HH | 853 | 3 | 0 | 0 | 4 | 846 |
|  | 0.01% SA | 753 | 5 | 1 | 3 | 10 | 734 |
|  | 0.02% SA | 579 | 1 | 0 | 0 | 5 | 573 |
| LPP 11100 | Parent | 40 | 0 | 0 | 0 | 1 | 39 |
|  | 0.1% EMS | 255 | 0 | 0 | 1 | 2 | 252 |
|  | 0.2% EMS | 54 | 0 | 0 | 0 | 0 | 54 |
|  | 0.02% HH | 358 | 1 | 0 | 0 | 2 | 255 |
|  | 0.03% HH | 254 | 3 | 1 | 1 | 5 | 244 |
|  | 0.01% SA | 231 | 5 | 0 | 1 | 6 | 219 |
|  | 0.02% SA | 215 | 0 | 0 | 1 | 5 | 209 |

^$^R = Resistant, T = Tolerant, MT = Moderately Tolerant, S = Sensitive and HS = Highly Sensitive

Table 6. Field reaction of M_2_ populations to ALLY MAX (1.5X dose) in screening through pre plant incorporation (PPI) method

| Genotype | Treatment | No. of plants studied | No. of plants with scoring of | | | | |
| --- | --- | --- | --- | --- | --- | --- | --- |
|  |  |  | 1 (R) | 2 (T) | 3 (MT) | 4 (S) | 5 (HS) |
| LPP 11001 | Parent | 40 | 0 | 0 | 1 | 3 | 36 |
|  | 0.1% EMS | 129 | 0 | 0 | 2 | 5 | 122 |
|  | 0.2% EMS | 40 | 0 | 0 | 1 | 2 | 37 |
|  | 0.02% HH | 345 | 0 | 0 | 3 | 3 | 339 |
|  | 0.03% HH | 258 | 1 | 0 | 2 | 6 | 249 |
|  | 0.01% SA | 111 | 1 | 0 | 1 | 3 | 106 |
|  | 0.02% SA | 143 | 5 | 1 | 3 | 4 | 130 |
| LPP 11116 | Parent | 40 | 1 | 1 | 2 | 1 | 35 |
|  | 0.1% EMS | 897 | 5 | 6 | 8 | 14 | 864 |
|  | 0.2% EMS | 318 | 2 | 4 | 5 | 4 | 302 |
|  | 0.02% HH | 773 | 6 | 6 | 4 | 19 | 738 |
|  | 0.03% HH | 853 | 3 | 12 | 6 | 10 | 822 |
|  | 0.01% SA | 753 | 1 | 2 | 5 | 8 | 737 |
|  | 0.02% SA | 579 | 0 | 1 | 3 | 9 | 566 |
| LPP 11100 | Parent | 40 | 0 | 0 | 1 | 2 | 37 |
|  | 0.1% EMS | 255 | 0 | 1 | 2 | 4 | 248 |
|  | 0.2% EMS | 54 | 0 | 0 | 1 | 3 | 50 |
|  | 0.02% HH | 358 | 1 | 0 | 4 | 3 | 350 |
|  | 0.03% HH | 254 | 0 | 1 | 2 | 6 | 245 |
|  | 0.01% SA | 231 | 0 | 1 | 1 | 3 | 226 |
|  | 0.02% SA | 215 | 0 | 0 | 0 | 5 | 210 |

^$^R = Resistant, T = Tolerant, MT = Moderately Tolerant, S = Sensitive and HS = Highly Sensitive

Table 7. Field reaction of M_2_ populations to ATLANTIS (X dose) in screening through pre plant incorporation (PPI) method

| Genotype | Treatment | No. of plants studied | No. of plants with scoring of | | | | |
| --- | --- | --- | --- | --- | --- | --- | --- |
|  |  |  | 1 (R) | 2 (T) | 3 (MT) | 4 (S) | 5 (HS) |
| LPP 11001 | Parent | 40 | 1 | 7 | 4 | 3 | 25 |
|  | 0.1% EMS | 129 | 6 | 12 | 9 | 9 | 93 |
|  | 0.2% EMS | 40 | 1 | 3 | 2 | 5 | 29 |
|  | 0.02% HH | 345 | 20 | 17 | 14 | 17 | 277 |
|  | 0.03% HH | 258 | 13 | 20 | 24 | 17 | 184 |
|  | 0.01% SA | 111 | 8 | 10 | 3 | 9 | 81 |
|  | 0.02% SA | 143 | 5 | 9 | 7 | 9 | 113 |
| LPP 11116 | Parent | 40 | 1 | 2 | 2 | 8 | 27 |
|  | 0.1% EMS | 897 | 20 | 11 | 6 | 82 | 778 |
|  | 0.2% EMS | 318 | 10 | 7 | 6 | 36 | 259 |
|  | 0.02% HH | 773 | 25 | 21 | 18 | 54 | 655 |
|  | 0.03% HH | 853 | 15 | 12 | 6 | 74 | 746 |
|  | 0.01% SA | 753 | 12 | 28 | 30 | 59 | 624 |
|  | 0.02% SA | 579 | 6 | 15 | 24 | 62 | 472 |
| LPP 11100 | Parent | 40 | 1 | 4 | 5 | 6 | 24 |
|  | 0.1% EMS | 255 | 4 | 8 | 11 | 87 | 145 |
|  | 0.2% EMS | 54 | 1 | 3 | 2 | 8 | 40 |
|  | 0.02% HH | 358 | 0 | 4 | 9 | 107 | 238 |
|  | 0.03% HH | 254 | 4 | 5 | 8 | 79 | 168 |
|  | 0.01% SA | 231 | 2 | 3 | 6 | 66 | 154 |
|  | 0.02% SA | 215 | 4 | 4 | 8 | 54 | 155 |

^$^R = Resistant, T = Tolerant, MT = Moderately Tolerant, S = Sensitive and HS = Highly Sensitive

Table 8. Field reaction of M_2_ populations to ATLANTIS (1.5X dose) in screening through pre plant incorporation (PPI) method

| Genotype | Treatment | No. of plants studied | No. of plants with scoring of | | | | |
| --- | --- | --- | --- | --- | --- | --- | --- |
|  |  |  | 1 (R) | 2 (T) | 3 (MT) | 4 (S) | 5 (HS) |
| LPP 11001 | Parent | 40 | 0 | 2 | 3 | 5 | 30 |
|  | 0.1% EMS | 129 | 1 | 4 | 3 | 10 | 111 |
|  | 0.2% EMS | 40 | 1 | 1 | 2 | 4 | 32 |
|  | 0.02% HH | 345 | 3 | 5 | 7 | 15 | 315 |
|  | 0.03% HH | 258 | 4 | 7 | 11 | 6 | 230 |
|  | 0.01% SA | 111 | 3 | 4 | 2 | 4 | 98 |
|  | 0.02% SA | 143 | 3 | 1 | 1 | 3 | 135 |
| LPP 11116 | Parent | 40 | 3 | 2 | 1 | 1 | 33 |
|  | 0.1% EMS | 897 | 7 | 5 | 7 | 19 | 859 |
|  | 0.2% EMS | 318 | 4 | 2 | 5 | 11 | 296 |
|  | 0.02% HH | 773 | 6 | 4 | 11 | 27 | 725 |
|  | 0.03% HH | 853 | 5 | 6 | 4 | 4 | 834 |
|  | 0.01% SA | 753 | 8 | 11 | 6 | 9 | 719 |
|  | 0.02% SA | 579 | 4 | 8 | 9 | 13 | 545 |
| LPP 11100 | Parent | 40 | 1 | 0 | 1 | 2 | 36 |
|  | 0.1% EMS | 255 | 2 | 2 | 11 | 9 | 231 |
|  | 0.2% EMS | 54 | 1 | 0 | 2 | 1 | 50 |
|  | 0.02% HH | 358 | 5 | 8 | 4 | 7 | 334 |
|  | 0.03% HH | 254 | 3 | 7 | 4 | 6 | 234 |
|  | 0.01% SA | 231 | 3 | 4 | 3 | 10 | 211 |
|  | 0.02% SA | 215 | 3 | 2 | 4 | 3 | 203 |

^$^R = Resistant, T = Tolerant, MT = Moderately Tolerant, S = Sensitive and HS = Highly Sensitive

Table 9. Field reaction of M_2_ populations to ALLY MAX (X dose) in screening through seed priming (SP) method

| Genotype | Treatment | No. of plants studied | No. of plants with scoring of | | | | |
| --- | --- | --- | --- | --- | --- | --- | --- |
|  |  |  | 1 (R) | 2 (T) | 3 (MT) | 4 (S) | 5 (HS) |
| LPP 11001 | Parent | 40 | 1 | 1 | 0 | 0 | 38 |
|  | 0.1% EMS | 129 | 0 | 1 | 0 | 1 | 127 |
|  | 0.2% EMS | 40 | 0 | 0 | 0 | 0 | 40 |
|  | 0.02% HH | 345 | 6 | 2 | 1 | 1 | 335 |
|  | 0.03% HH | 258 | 4 | 2 | 0 | 3 | 249 |
|  | 0.01% SA | 111 | 1 | 0 | 0 | 1 | 109 |
|  | 0.02% SA | 143 | 0 | 0 | 0 | 2 | 141 |
| LPP 11116 | Parent | 40 | 1 | 0 | 2 | 0 | 37 |
|  | 0.1% EMS | 897 | 0 | 0 | 1 | 8 | 888 |
|  | 0.2% EMS | 318 | 0 | 0 | 1 | 5 | 312 |
|  | 0.02% HH | 773 | 6 | 3 | 3 | 8 | 753 |
|  | 0.03% HH | 853 | 0 | 2 | 0 | 2 | 849 |
|  | 0.01% SA | 753 | 7 | 1 | 4 | 13 | 728 |
|  | 0.02% SA | 579 | 1 | 0 | 0 | 6 | 572 |
| LPP 11100 | Parent | 40 | 0 | 0 | 0 | 0 | 40 |
|  | 0.1% EMS | 255 | 0 | 0 | 1 | 1 | 253 |
|  | 0.2% EMS | 54 | 0 | 0 | 0 | 0 | 54 |
|  | 0.02% HH | 358 | 0 | 1 | 1 | 2 | 354 |
|  | 0.03% HH | 254 | 0 | 0 | 0 | 2 | 252 |
|  | 0.01% SA | 231 | 2 | 0 | 0 | 6 | 223 |
|  | 0.02% SA | 215 | 2 | 0 | 1 | 2 | 210 |

^$^R = Resistant, T = Tolerant, MT = Moderately Tolerant, S = Sensitive and HS = Highly Sensitive

Table 10. Field reaction of M_2_ populations to ALLY MAX (1.5X dose) in screening through seed priming (SP) method

| Genotype | Treatment | No. of plants studied | No. of plants with scoring of | | | | |
| --- | --- | --- | --- | --- | --- | --- | --- |
|  |  |  | 1 (R) | 2 (T) | 3 (MT) | 4 (S) | 5 (HS) |
| LPP 11001 | Parent | 40 | 0 | 0 | 1 | 0 | 39 |
|  | 0.1% EMS | 129 | 0 | 0 | 2 | 0 | 127 |
|  | 0.2% EMS | 40 | 0 | 0 | 0 | 0 | 40 |
|  | 0.02% HH | 345 | 1 | 1 | 5 | 4 | 334 |
|  | 0.03% HH | 258 | 2 | 1 | 0 | 3 | 252 |
|  | 0.01% SA | 111 | 1 | 0 | 0 | 0 | 110 |
|  | 0.02% SA | 143 | 0 | 0 | 0 | 0 | 143 |
| LPP 11116 | Parent | 40 | 0 | 0 | 0 | 1 | 39 |
|  | 0.1% EMS | 897 | 1 | 0 | 2 | 10 | 884 |
|  | 0.2% EMS | 318 | 1 | 0 | 3 | 4 | 310 |
|  | 0.02% HH | 773 | 5 | 3 | 2 | 5 | 758 |
|  | 0.03% HH | 853 | 1 | 1 | 3 | 2 | 846 |
|  | 0.01% SA | 753 | 2 | 1 | 3 | 5 | 742 |
|  | 0.02% SA | 579 | 1 | 0 | 0 | 3 | 575 |
| LPP 11100 | Parent | 40 | 0 | 0 | 0 | 0 | 40 |
|  | 0.1% EMS | 255 | 0 | 2 | 0 | 1 | 252 |
|  | 0.2% EMS | 54 | 0 | 0 | 1 | 0 | 53 |
|  | 0.02% HH | 358 | 1 | 2 | 1 | 0 | 354 |
|  | 0.03% HH | 254 | 2 | 1 | 0 | 2 | 249 |
|  | 0.01% SA | 231 | 0 | 1 | 2 | 2 | 226 |
|  | 0.02% SA | 215 | 1 | 0 | 1 | 1 | 212 |

^$^R = Resistant, T = Tolerant, MT = Moderately Tolerant, S = Sensitive and HS = Highly Sensitive

Table 11. Field reaction of M_2_ populations to ATLANTIS (X dose) in screening through seed priming (SP) method

| Genotype | Treatment | No. of plants studied | No. of plants with scoring of | | | | |
| --- | --- | --- | --- | --- | --- | --- | --- |
|  |  |  | 1 (R) | 2 (T) | 3 (MT) | 4 (S) | 5 (HS) |
| LPP 11001 | Parent | 40 | 1 | 2 | 0 | 0 | 37 |
|  | 0.1% EMS | 129 | 1 | 0 | 0 | 0 | 128 |
|  | 0.2% EMS | 40 | 0 | 0 | 0 | 0 | 40 |
|  | 0.02% HH | 345 | 1 | 0 | 2 | 2 | 340 |
|  | 0.03% HH | 258 | 4 | 3 | 4 | 2 | 245 |
|  | 0.01% SA | 111 | 0 | 0 | 0 | 1 | 110 |
|  | 0.02% SA | 143 | 1 | 0 | 0 | 1 | 141 |
| LPP 11116 | Parent | 40 | 1 | 1 | 1 | 1 | 36 |
|  | 0.1% EMS | 897 | 1 | 0 | 1 | 10 | 885 |
|  | 0.2% EMS | 318 | 1 | 0 | 2 | 6 | 309 |
|  | 0.02% HH | 773 | 7 | 9 | 2 | 5 | 750 |
|  | 0.03% HH | 853 | 0 | 0 | 0 | 4 | 849 |
|  | 0.01% SA | 753 | 2 | 3 | 3 | 4 | 741 |
|  | 0.02% SA | 579 | 0 | 0 | 1 | 1 | 577 |
| LPP 11100 | Parent | 40 | 0 | 0 | 0 | 0 | 40 |
|  | 0.1% EMS | 255 | 1 | 0 | 0 | 1 | 253 |
|  | 0.2% EMS | 54 | 0 | 0 | 0 | 0 | 54 |
|  | 0.02% HH | 358 | 1 | 1 | 2 | 1 | 353 |
|  | 0.03% HH | 254 | 1 | 1 | 2 | 2 | 248 |
|  | 0.01% SA | 231 | 2 | 0 | 1 | 3 | 225 |
|  | 0.02% SA | 215 | 1 | 0 | 3 | 2 | 209 |

^$^R = Resistant, T = Tolerant, MT = Moderately Tolerant, S = Sensitive and HS = Highly Sensitive

Table 12. Field reaction of M_2_ populations to ATLANTIS (1.5X dose) in screening through seed priming (SP) method

| Genotype | Treatment | No. of plants studied | No. of plants with scoring of | | | | |
| --- | --- | --- | --- | --- | --- | --- | --- |
|  |  |  | 1 (R) | 2 (T) | 3 (MT) | 4 (S) | 5 (HS) |
| LPP 11001 | Parent | 40 | 2 | 1 | 2 | 2 | 33 |
|  | 0.1% EMS | 129 | 0 | 2 | 0 | 3 | 124 |
|  | 0.2% EMS | 40 | 0 | 0 | 0 | 3 | 37 |
|  | 0.02% HH | 345 | 0 | 0 | 2 | 13 | 330 |
|  | 0.03% HH | 258 | 0 | 0 | 0 | 1 | 257 |
|  | 0.01% SA | 111 | 0 | 0 | 0 | 0 | 111 |
|  | 0.02% SA | 143 | 0 | 1 | 0 | 7 | 135 |
| LPP 11116 | Parent | 40 | 0 | 0 | 0 | 5 | 35 |
|  | 0.1% EMS | 897 | 2 | 1 | 1 | 21 | 872 |
|  | 0.2% EMS | 318 | 0 | 0 | 0 | 5 | 313 |
|  | 0.02% HH | 773 | 0 | 1 | 2 | 23 | 747 |
|  | 0.03% HH | 853 | 0 | 1 | 2 | 7 | 843 |
|  | 0.01% SA | 753 | 19 | 34 | 6 | 33 | 661 |
|  | 0.02% SA | 579 | 1 | 0 | 5 | 8 | 565 |
| LPP 11100 | Parent | 40 | 0 | 0 | 0 | 0 | 40 |
|  | 0.1% EMS | 255 | 0 | 0 | 0 | 1 | 254 |
|  | 0.2% EMS | 54 | 0 | 0 | 0 | 0 | 54 |
|  | 0.02% HH | 358 | 0 | 0 | 0 | 1 | 357 |
|  | 0.03% HH | 254 | 0 | 1 | 5 | 4 | 244 |
|  | 0.01% SA | 231 | 0 | 0 | 0 | 1 | 230 |
|  | 0.02% SA | 215 | 0 | 0 | 0 | 1 | 214 |

^$^R = Resistant, T = Tolerant, MT = Moderately Tolerant, S = Sensitive and HS = Highly Sensitive
